# Biosynthesis of Ephedrine Initiated by Pyridoxal Phosphate‐Dependent Formation of Cathinone

**DOI:** 10.1002/cbic.202500279

**Published:** 2025-06-23

**Authors:** Karina Witte, Anne Behrens, Hannes M. Schwelm, Volker Auwärter, Michael Müller

**Affiliations:** ^1^ Institute of Pharmaceutical Sciences University of Freiburg Albertstrasse 25 79104 Freiburg Germany; ^2^ Institute of Forensic Medicine Forensic Toxicology Medical Faculty ‐ University of Freiburg 79104 Freiburg Germany

**Keywords:** biosyntheses, ephedrines, pyridoxal phosphates

## Abstract

Ephedra alkaloids possess some of the most basic structures of alkaloids. Despite their importance for human use and their commercial relevance, the biosynthesis of ephedra alkaloids has remained enigmatic. The predominant biosynthetic pathway in the literature proposes a thiamin‐dependent carboligation followed by a transaminase, although no candidate enzymes have yet been identified in ephedra alkaloid producers. In this work, an alternative pathway in plants to ephedra alkaloids via (*S*)–cathinone is investigated that circumvents the formation of 1‐phenylpropane‐1,2‐dione as an intermediate and is in full agreement with previous biosynthetic studies. This alternative pathway involves the pyridoxal phosphate (PLP)‐dependent carboligation of –benzoyl‐CoA– and l‐alanine in a single step. The PLP‐dependent formation of labeled and unlabeled (*S*)–cathinone is detected in the plant lysate of young stem tissue of various *Ephedra* species that contain Ephedra alkaloids, as well as in young leaf tissue of *Catha edulis*. The incorporation of labeled nitrogen from l‐alanine into (*S*)‐cathinone supports the hypothesis that an α‐oxoamine synthase (AOS) catalyzes the formation of (*S*)‐cathinone, bypassing the dione as an intermediate. These results demonstrate the involvement of a PLP‐dependent AOS as a pivotal step in the biosynthesis of ephedra alkaloids.

## Graphical abstract

An alternative pathway to ephedra alkaloids via (*S*)–cathinone is investigated in plants. This pathway circumvents the formation of 1‐phenylpropane‐1,2‐dione as an intermediate, which has been part of the predominantly postulated biosynthetic pathway for the last decades. This alternative pathway involves an enzymatic pyridoxal phosphate (PLP)‐dependent carboligation of benzoyl‐CoA and l‐alanine in a single step.

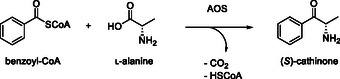

## 1 Introduction

Ephedra alkaloids represent an important group of alkaloids such as cathinone (**1**), nor(pseudo)ephedrine (**2**), and (pseudo)ephedrine (**3**). They share a 1–phenylpropan–2–amine core structure and contain an α–oxyamine structure as a building block, which is widely present in a range of indirect sympathomimetic drugs for medicinal and recreational purposes.^[^ ^1^ ^]^ Ephedra alkaloids have been isolated from plants such as *Ephedra* species^[^ ^2^ ^]^ and *Catha edulis*,^[^ ^3^ ^]^ and their biosynthesis has been studied for more than six decades using a variety of approaches, including labeled compound incorporation studies and identification and characterization of the enzymes involved.^[^ ^1^, ^4^, ^5^, ^6^, ^7^ ^]^ However, major intermediates and enzymes remain elusive. Only a SAM‐dependent N‐methyltransferase (PaNMT) has been identified as being involved in the biosynthesis of ephedrine.^[^ ^4^ ^]^ In particular, the formation of **1** as the initial ephedra alkaloid in biosynthesis requires further investigation.^[^ ^4^, ^8^ ^]^

**Scheme 1:**
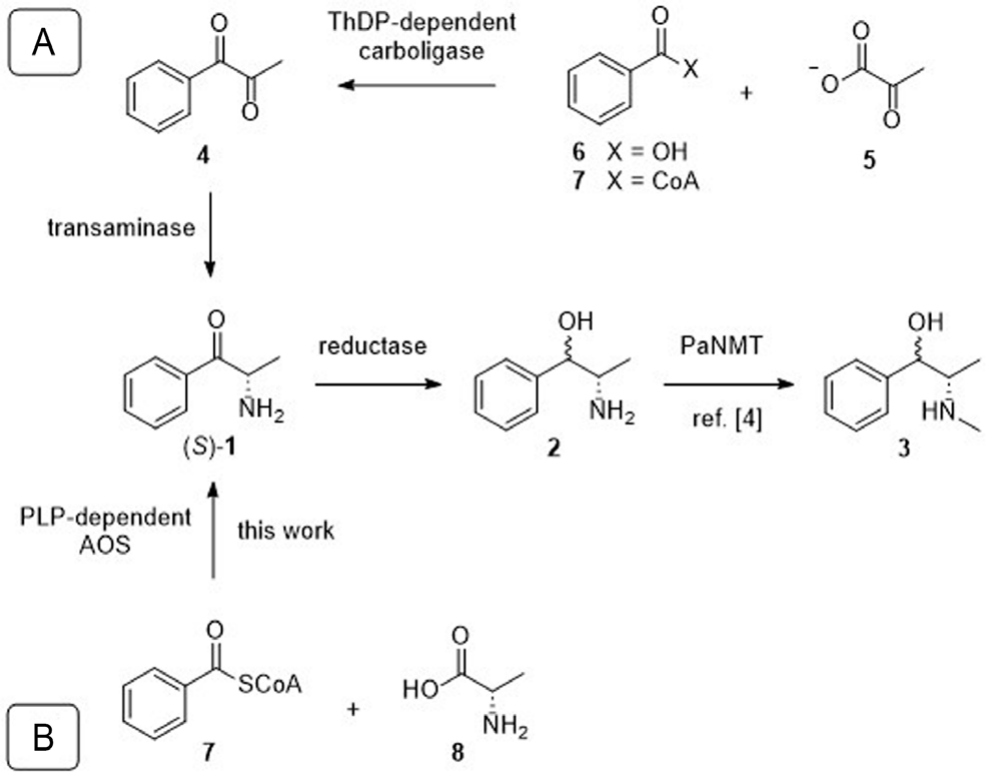
A) Predominantly postulated biosynthesis in literature: ThDP‐dependent carboligation of pyruvate (**5**) with benzoic acid (**6**) or benzoyl‐CoA (**7**) would result in dione **4**, which is to be transaminated to cathinone [(*S*)‐**1**]. B) This work: alternative PLP‐dependent biosynthesis to (*S*)‐1. Both pathways have in common that (*S*)–**1** undergoes reduction to nor(pseudo)ephedrine (**2**) and methylation to (pseudo)ephedrine (**3**); PaNMT: phenylalkylamine N‐methyltransferase.^[^ ^1^, ^4^ ^]^

Thus far, the prevailing hypothesis is that the formation of **1** occurs by transamination from 1‐phenylpropane‐1,2‐dione (**4**). Grue–Sørensen and Spenser postulated dione **4** as a precursor of ephedra alkaloids formed by a thiamin diphosphate (ThDP)‐dependent carboligation of pyruvate (**5**) with benzoic acid (**6**) or benzoyl‐CoA (**7**) (**Scheme** **1**, route A). Thus, **6** or **7** would be the precursor of the phenyl ring and C1, and **5** the precursor of C2 and C3.^[^ ^9^, ^10^ ^]^ Moreover, labeling experiments with [^13^C_1,2,3_]–**4** supposedly showed incorporation into norephedrine [(1* R*,2*S*)–**2**] and norpseudoephedrine [(1*S*,2*S*)–**2**]. Incorporation of the label into pseudoephedrine [(1*S*,2*S*)–**3**] was low and undetectable for ephedrine [(1* R*,2*S*)–**3**]. Small amounts of labeled (*S*)–**1** were detected.^[^ ^9^, ^10^ ^]^ Krizevski et al. detected **4** and (*S*)‐**1** in GC–MS analysis of freshly harvested young *E. sinica* stems. However, they did not detect the carboligation reaction.^[^ ^11^ ^]^ Furthermore, due to its reactivity, **4** can also be degraded or nonenzymatically transaminated to (*S*)–**1**, which can be converted to the other ephedra alkaloids. Despite tremendous efforts, the putative ThDP‐dependent carboligase and transaminase have not been identified in plants as yet.^[^ ^1^, ^4^ ^]^

**Scheme 2:**
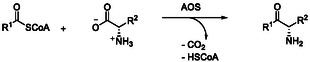
General reaction catalyzed by putative α‐oxoamine synthases (AOS), which form an α–amino ketone structure via a Claisen‐like condensation.

α–Oxyamine moieties can be formed via different biosynthetic or biocatalytic pathways. One of these pathways is via α‐oxoamine synthases (AOS), which are pyridoxal 5‘–phosphate (PLP)‐dependent enzymes that catalyze the formation of α–amino ketones via a Claisen–like condensation. In this process, a new C—C bond is formed by decarboxylative coupling of amino acids with α–keto thioesters such as –acyl‐CoA– (**Scheme** **2**).^[^ ^12^, ^13^ ^]^

We investigated this alternative pathway to (*S*)–**1** catalyzed by an AOS, hypothesized to be involved in the biosynthesis of ephedra alkaloids (Scheme 1, route B). This pathway involves the PLP‐dependent– carboligation of –benzoyl‐CoA– (**7**) and l–alanine– (**8**) in one step, avoiding the formation of dione **4** as an intermediate.

An HPLC‐MS/MS‐based assay was developed to test AOS activity in plants. Plant samples of young tissue of *Catha edulis* and 14 different *Ephedra* species were collected from botanical gardens and analyzed for their ephedra alkaloid content and AOS activity. The young plant tissue of each plant was freshly harvested, lysed with a bead homogenizer, and centrifuged. The supernatant of the lysate was purified from the ephedra alkaloids contained in the plant lysate by size exclusion chromatography. The flow‐through was analyzed as a blank for each assay to confirm complete removal of the small molecules from the plant lysate. To distinguish between enzymatic and nonenzymatic product formation, negative control reactions were performed with lysate heat inactivated at 95 °C for 20 min. The dependence of the conversions on PLP was evaluated using plant lysate with or without PLP after removal of PLP by size exclusion chromatography. Subsequently, the PLP‐free plant lysate was subjected to further tests after the reintroduction of PLP.

**Scheme 3:**
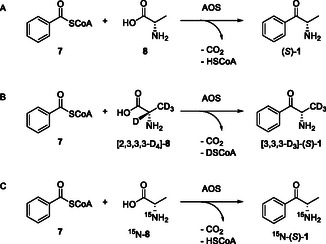
Detected conversions of benzoyl‐CoA (**7**) and (un)labeled l‐alanine (**8**) to (*S*)–cathinone [(*S*)–**1**] catalyzed by a putative α‐oxoamine synthase (AOS) in lysates of various *Ephedra* species and *Catha edulis*. A) unlabeled **8**; B) [2,3,3,3‐D_4_]‐**8**; C) ^15^ N‐**8**.

The formation of (*S*)–**1** was detected in the plant lysate of young stem tissue of all *Ephedra* species that contained ephedra alkaloids and in *Catha edulis*. No formation or only traces of (*S*)–**1** were detected in the plant lysate of young stem tissue of *Ephedra* species that did not contain ephedra alkaloids. To confirm the formation of (*S*)–**1** during the assay via an AOS mechanism, deuterium‐labeled l‐alanine ([2,3,3,3–D_4_]‐**8**) was used, and the formation of (*S*)–[3,3,3–D_3_]–**1** was detected (**Figure** **1**C, **Scheme** **3**B).

Figure 1HPLC‐MS/MS chromatograms of the three activity assays performed on the purified lysate of *Catha edulis* collected in May 2022 at the Botanical Garden of Bern, Switzerland. The chromatograms show the isolated ion transitions from the MRM scans expected for (*S*)–1 (left), (*S*)–[3,3,3–D_3_]–1 (center), and (*S*)–^15^ N–1 (right). A) Lysate as blank; B) activity assay with 7 and 8; C) activity assay with 7 and [2,3,3,3–D_4_]–8; and D) activity assay with 7 and ^15^ N–8. The peak at 24 min represents the (*S*)‐isomer and the peak at 30 min represents the (*R*)‐isomer.

**Figure:**
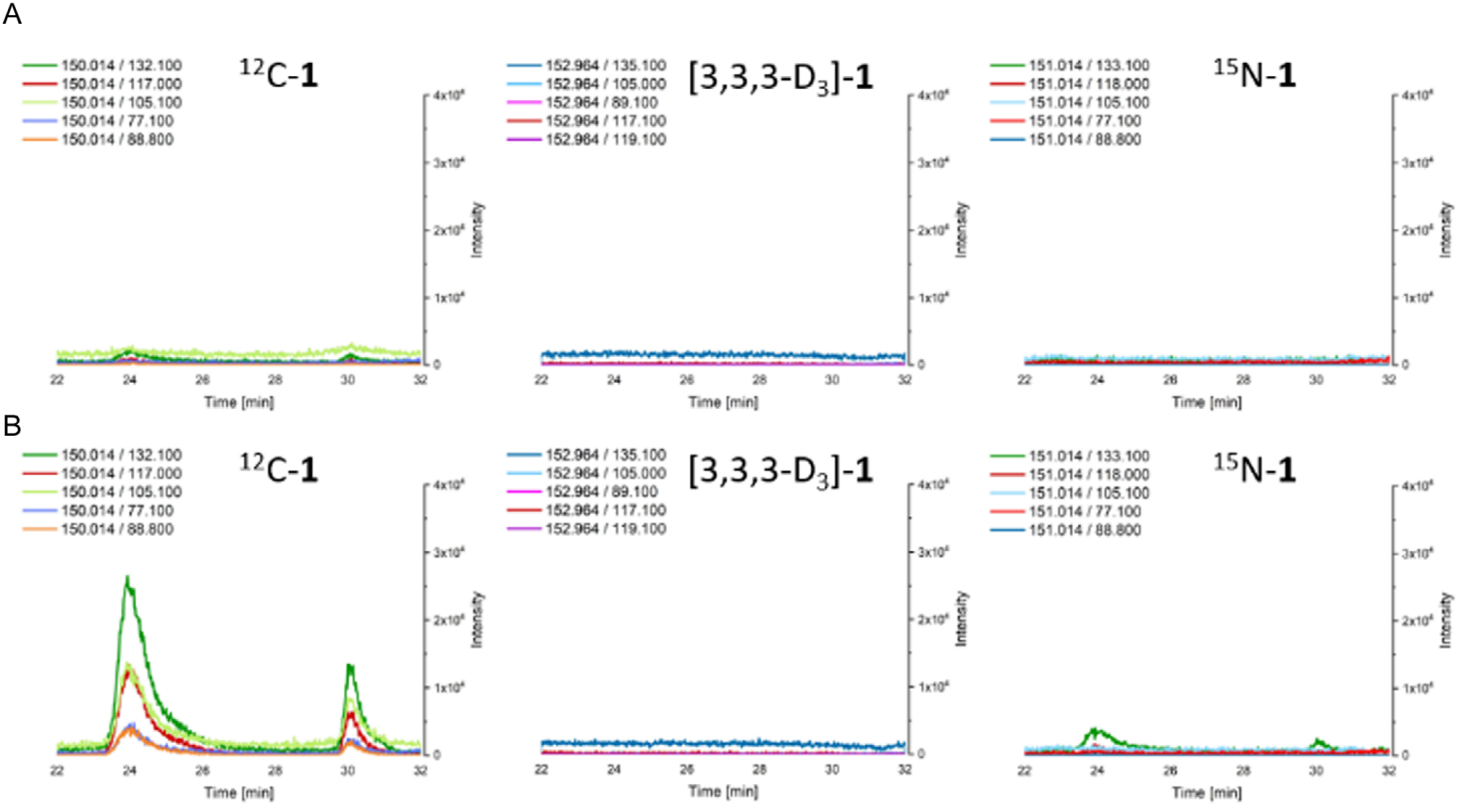

**Figure:**
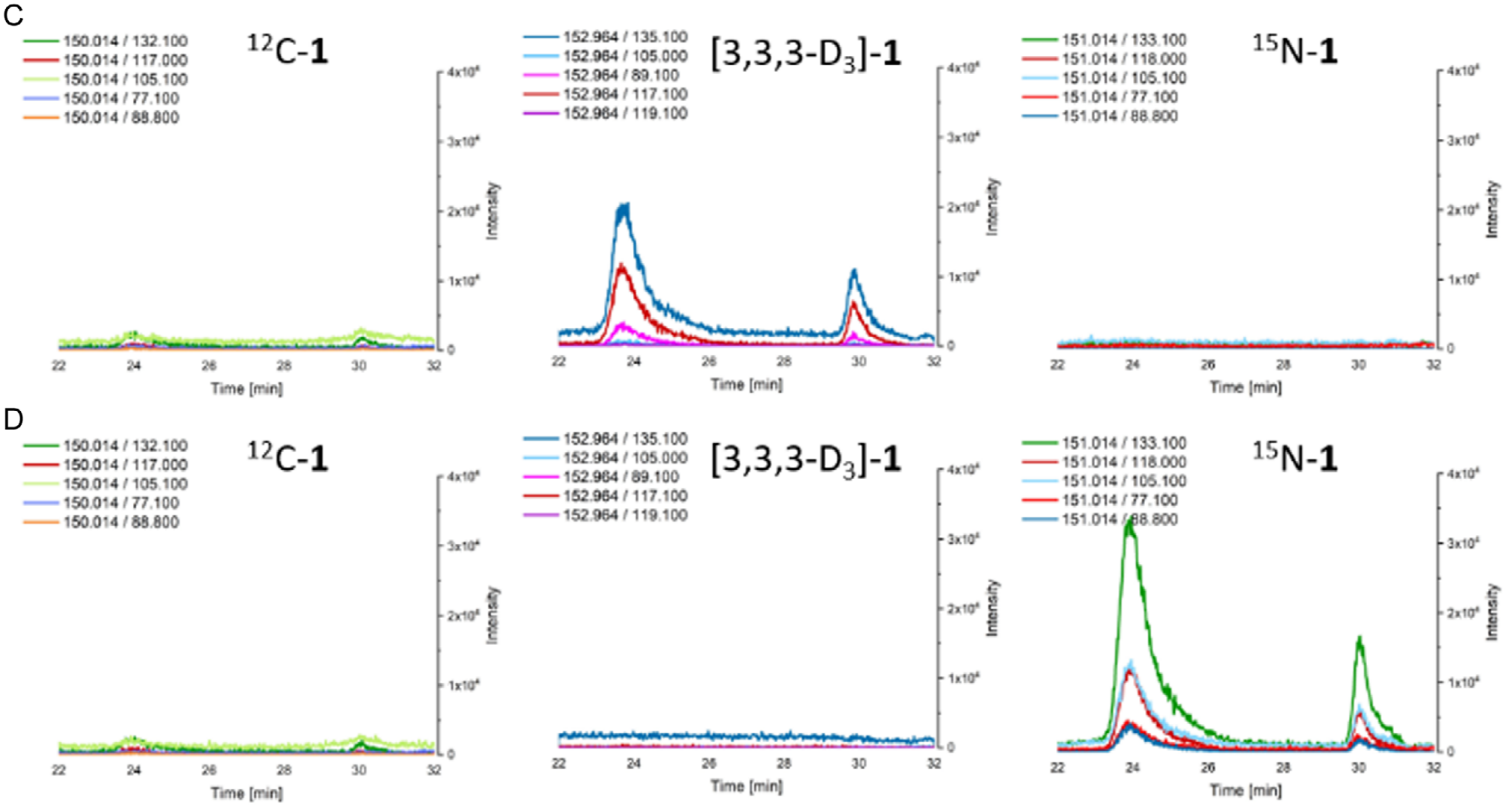

Nevertheless, the formation of (*S*)–[3,3,3–D_3_]–**1** upon addition of **7** and [2,3,3,3–D_4_]–**8** to an enzyme or plant lysate is not firm evidence for the proposed reaction mechanism by an AOS. Krizevski et al. postulated that benzaldehyde is a precursor of phenylpropylamino alkaloids. In their studies, they show a ThDP‐dependent carboligase activity in soluble protein preparations from *E. sinica* and *E. foeminea* stems, which catalyze the conversion of benzaldehyde and pyruvate to (*R*)– and (*S*)–phenylacetylcarbinol, (*R*)– and (*S*)–2–hydroxypropiophenone, and dione **4**.^[^ ^1^ ^]^ Hence, [2,3,3,3–D_4_]–**8** could undergo transamination to [3,3,3–D_3_]–pyruvate [[3,3,3–D_3_]–**5**], followed by conversion to [3,3,3–D_3_]–**4** catalyzed by a ThDP‐dependent carboligase. The dione **4** could be converted via a transaminase to (*S*)–[3,3,3–D_3_]–**1** (see Supporting Information), which would be consistent with the biosynthetic pathway postulated in the literature.

To investigate the mechanism of (*S*)–**1** formation and to confirm beyond doubt the conversion of **7** and **8** by an AOS, ^15^N‐labeled **8** was used as a substrate for the AOS activity assay. In the transaminase mechanism, the nitrogen is removed from **8**, whereas in our experiments, labeled nitrogen remains in the molecule and is incorporated into the product (*S*)–^15^N–**1** (Figure 1D, Scheme 3C), which is in agreement with the postulated reaction mechanism by an AOS.

**Scheme 4:**
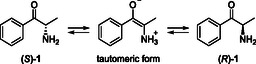
Possible tautomerization of (*S*)‐cathinone [(*S*)‐**1**].

In addition to the formation of (*S*)‐**1**, the presence of (*R*)‐**1** was detected in all activity assays as well as the analyses of all plant lysates. Krizevski et al. describe the detection of (*S*)‐ and (*R*)‐**1** in their analyses of *Catha edulis,* which they explain by the possible partial ketoamino tautomerization of (*S*)–**1** under their method conditions (**Scheme** **4**).^[^ ^14^ ^]^ The putative involvement of (*R*)‐**1** should be investigated in future studies.

Experiments were conducted to analyze the PLP dependency of the putative AOS involved in the detected reaction. Activity assays were performed with plant lysate from *E. gerardiana* and *E. americana*. The results showed that no (*S*)–**1** formation occurred when PLP was removed via size exclusion chromatography. Conversely, (*S*)–**1** was formed when PLP was added back to the reaction mixture, indicating a PLP‐dependent reaction mechanism.

The results of this study demonstrate the existence of a new PLP‐dependent enzymatic activity in *Ephedra* species producing ephedra alkaloids and in *C. edulis*. The incorporation of the deuterium label of [2,3,3,3‐D_4_]–**8** and the nitrogen label of ^15^N‐**8** into (*S*)–**1** was demonstrated by labeling experiments (see Figure 1 and Scheme 3). In particular, the incorporation of the nitrogen label and the PLP‐dependency of (*S*)‐**1** formation supports the hypothesis that an AOS catalyzes the formation of **1** and bypasses the need for dione **4** as an intermediate.

The identification of the AOS enzyme responsible for the PLP‐dependent formation of (*S*)‐**1** in *Ephedra* species is ongoing and crucial for further testing of our hypothesis; so far, it has been hampered by the many PLP‐dependent enzymes in the plants.

## Conflict of Interest

The authors declare no conflict of interest.

## Supporting information

Supplementary Material

## Data Availability

The data that support the findings of this study are available in the supplementary material of this article.

## References

[1] J. M. Hagel , R. Krizevski , F. Marsolais , E. Lewinsohn , P. J. Facchini , Trends Plant Sci. 2012, 17, 404. 22502775 10.1016/j.tplants.2012.03.004

[2] N. A. O’Dowd , P. G. McCauley , G. Wilson , J. A. N. Parnell , T. A. K. Kavanagh , D. J. McConnell 1998, 41, 154‐193.

[3] X. Schorno , E. Steinegger , Experientia 1979, 35, 572.

[4] J. S. Morris , R. A. Groves , J. M. Hagel , P. J. Facchini , J. Biol. Chem. 2018, 293, 13364. 29929980 10.1074/jbc.RA118.004067 PMC6120201

[5] E. Leete , Science 1965, 147, 1000. 14245774 10.1126/science.147.3661.1000

[6] K. Yamasaki , U. Sankawa , S. Shibata , Tetrahedron Lett. 1969, 10, 4099.

[7] G. Grue‐Sørensen , I. D. Spenser , Can. J. Chem. 1989, 67, 998.

[8] R. A. Groves , J. M. Hagel , Y. Zhang , K. Kilpatrick , A. Levy , F. Marsolais , E. Lewinsohn , C. W. Sensen , P. J. Facchini , PloS One 2015, 10, e0119701. 25806807 10.1371/journal.pone.0119701 PMC4373857

[9] G. Grue‐Soerensen , I. D. Spenser , J. Am. Chem. Soc. 1993, 115, 2052.

[10] G. Grue‐Soerensen , I. D. Spenser , J. Am. Chem. Soc. 1994, 116, 6195.

[11] R. Krizevski , E. Bar , O. Shalit , Y. Sitrit , S. Ben‐Shabat , E. Lewinsohn , Phytochemistry 2010, 71, 895. 20417943 10.1016/j.phytochem.2010.03.019

[12] O. Ploux , A. Marquet , Eur. J. Biochem. 1996, 236, 301. 8617279 10.1111/j.1432-1033.1996.00301.x

[13] D. Alexeev , M. Alexeeva , R. L. Baxter , D. J. Campopiano , S. P. Webster , L. Sawyer , J. Mol. Biol. 1998, 284, 401. 9813126 10.1006/jmbi.1998.2086

[14] R. Krizevski , N. Dudai , E. Bar , I. Dessow , U. Ravid , E. Lewinsohn , Isr. J. Plant Sci. 2008, 56, 207.

