# Supplementary material for: Biosynthesis of Ephedrine Initiated by Pyridoxal Phosphate‐Dependent Formation of Cathinone
Source: Chembiochem. 2025 Jun 23;26(14):e202500279. doi: 10.1002/cbic.202500279 (PMC12278337; doi:10.1002/cbic.202500279)
Supplement: Supplementary file 1 — Supplementary Material [file CBIC-26-e202500279-s001.pdf]

Supporting Information

Biosynthesis of Ephedrine is Initiated by Pyridoxal Phosphate-Dependent Formation of Cathinone

Karina Witte,[a] Anne Behrens,[a] Hannes M. Schwelm,[b] Volker Auwärter,[b] Michael Müller[a]*

[a] Institute of Pharmaceutical Sciences, University of Freiburg,
Albertstrasse 25, 79104 Freiburg, Germany
E-mail: michael.mueller@pharmazie.uni-freiburg.de

[b] Institute of Forensic Medicine, Forensic Toxicology,
Medical Center - University of Freiburg, 79104 Freiburg, Germany

1 General Remarks 2
2 Fragmentation Pattern of (*S*)-Cathinone 9
3 Possible Incorporation of Deuterium Label via Transamination 11
4 Ephedra Alkaloid Content in tested Plants 12
5 Formation of (*S*)-1 in Activity Assay 15
6 PLP-dependence of (*S*)-Cathinone Formation in Plant Lysate 17

1 General Remarks

1.1 High-Performance Liquid Chromatography-Tandem Mass Spectrometry

High-Performance Liquid Chromatography-Tandem Mass Spectrometry (HPLC-MS/MS) analysis was performed using an 1100 Series HPLC system (Agilent, Santa Clara, USA), coupled with a QTRAP® 4500 mass spectrometer (AB SCIEX, Darmstadt, Germany) and electrospray ionization (ESI).

For enantiomeric separation, a chiral Lux 3 µm AMP® column (150 x 3 mm, Phenomenex, Torrance, USA) was used equipped with a guard column (SecurityGuard™ ULTRA cartridge, Phenomenex, Torrance, USA). The injection volume for HPLC analysis was 5 µL and the solvents and methods are listed in Table S1 and S2. Mass spectrometry was conducted in MRM scan mode, parameters are listed in Table S3, and all isolated ions are shown in Table S4.

Table S1. Composition of solvents for chiral HPLC-MS/MS analysis.

	Component	Contents/Volumes
Solvent A (pH 11.0)	NH_4HCO_3	395 mg
	NH_3 (aq)	pH adjustment
	$_{dd}H_2O$	to 1.0 L
Solvent B	Methanol	560 mL
	Acetonitrile	280 mL
	2-propanol	140 mL

Table S2. HPLC method for chiral HPLC-MS/MS analysis.

Time [min]	Solvent B [%]
0.0	32.5
20.0	42.5
25.0	95.0
29.0	95.0
32.0	32.5
46.0	32.5

**Flow [mL·min^{-1}]**	0.20

Table S3. MRM scan parameters for chiral HPLC-MS/MS analysis.

**Parameter**	**Value**
Polarity	Positive
Curtain gas (CUR)	30 psi
Temperature (TEM)	550 °C
Gas 1 (GS1)	60 psi
Gas 2 (GS2)	60 psi
Entrance potential (EP)	10 V
Collision gas (CAD)	High
IonSpray voltage (IS)	4000 V

Table S4. Ions isolated in MRM scans for chiral HPLC-MS/MS analysis.

**Compound**	**Q1 (*m*/*z*)**	**Q3 (*m*/*z*)**
Cathinone[a]	150.014	132.100
	150.014	117.000
	150.014	105.100
	150.014	88.800
	150.014	77.100
[3,3,3-D_3]-Cathinone[b]	152.964	135.100
	152.964	117.100
	152.964	89.100
	152.964	105.000
	152.964	119.100
^{15}N-Cathinone[c]	151.014	133.100
	151.014	118.000
	151.014	105.100
	151.014	88.800
	151.014	77.100
Nor(pseudo)ephedrine[a]	151.749	133.800
	151.749	117.100

	151.749	115.100
	151.749	90.900
(Pseudo)ephedrine[a]	166.140	148.100
	166.140	115.000
	166.140	133.200
	166.140	117.100
	166.140	91.000
N-Methyl(pseudo)ephedrine[a]	180.241	162.000
	180.241	91.100
	180.241	115.100
	180.241	46.000
	180.241	42.900
N-Methylcathinone[a]	164.007	146.100
	164.007	131.200
	164.007	130.100
	164.007	76.900
	164.007	104.900

[a] method optimized using references;
[b] method manually adapted to (*m*/z + 3);
[c] method manually adapted to (*m*/z + 1).

Table S5. Assignment of Ephedra alkaloids to the peaks observed in the chiral HPLC-MS/MS method.

Peak	Retention Time [min]	Assigned *Ephedra* Alkaloid		
		Name	Identification	Structure
1	12.22	(1*S*,2*R*)-Norephedrine	Assigned	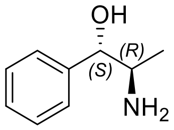
2	14.46	(1*S*,2*S*)-Norpseudoephedrine	Standard reference	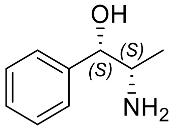
3	18.37	(1*R*,2*S*)-Ephedrine	Standard reference	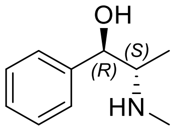
4	21.32	(1*S*,2*S*)-Pseudoephedrine	Assigned	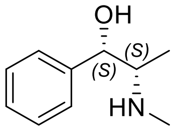
5	24.29	(1*R*,2*S*)-Norephedrine	Assigned	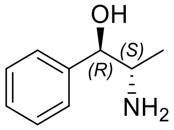
6	24.79	(1*R*,2*S*)-*N*-Methylephedrine	Standard reference	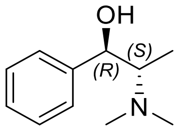
7	25.39	(*S*)-Cathinone	Standard reference	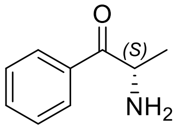
8	29.39	(*R*/*S*)-*N*-Methylcathinone	Racemic standard reference	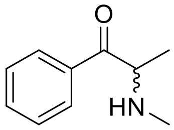
9	30.60	(*S*/*R*)-*N*-Methylcathinone	Racemic standard reference	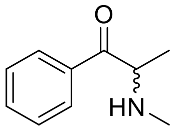
10	31.52	(*R*)-Cathinone	Assigned	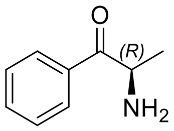
11	31.82	(1*S*,2*S*)-*N*-Methylpseudoephedrine	Assigned	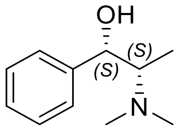

1.2 Plant Alkaloid and Protein Purification

1.2.1 Cell Disruption

Plant material was freshly picked or thawed from –80 °C storage before use. Lysis was performed with a bead homogenizer. The composition of buffers used for lysis is shown in Table S6. Fresh plant material (1 g) was mixed with 0.1 g PVPP in 3 mL of lysis buffer (Table S6). If lysate was intended for use in activity assays, 0.1 mL Plant Protease Inhibitors (Merck, Darmstadt, Germany) was added. Lysis was conducted using a Precellys homogenizer (Peqlab, Erlangen, Germany) with a Precellys Ceramic Kit (2.8mm, 7 mL) for 5–6 times at 6000 rpm and 20 s with 2 min breaks on ice. The crude lysate was transferred into a new flask, washed with 1.5 mL of lysis buffer, and centrifuged at 8228 × *g* and 4 °C for 40 min. The supernatant of the lysate was directly used for purification, extraction, or activity assays.

Table S6. Composition of buffers used for lysis of plant samples.

	Component	Contents/Volumes
	TRIS HCl	15.76 g
	Glycerol	126 g
	PVP-40	10 g
Lysis buffer A (pH 7.5)	Dithiothreitol	771 mg
	PLP	27.7 mg
	KOH	pH adjustment
	$_{dd}H_2O$	to 1.0 L
	TRIS HCl	15.76 g
	PVP-40	10 g
Lysis buffer B (pH 7.5)	PLP	27.7 mg
	KOH	pH adjustment
	$_{dd}H_2O$	to 1.0 L
	TRIS HCl	15.76 g
	PVP-40	10 g
Lysis buffer C (pH 7.5)	KOH	pH adjustment
	$_{dd}H_2O$	to 1.0 L

1.2.2 Size Exclusion Chromatography

Size exclusion chromatography (SEC) was performed with PD-10 columns (Cytiva, Buckinghamshire, UK) following the gravity protocol for an aqueous solution. Columns were equilibrated with 25 mL of lysis buffer (Table S6) and loaded with 2.5 mL of lysate. Flowthrough was discarded before adding 3.5 mL of ice-cold buffer for elution. As a change from the standard protocol, only 2.5 mL of flowthrough was collected to avoid the remaining small molecules of plant lysate. The desalted lysate was purified a second time via SEC or directly used for activity assays without further storage.

1.2.3 Alkaloid Extraction

Method A

For alkaloid extraction via method A, the pH of 200 µL of lysate was adjusted using 10 µL of NaOH (0.1 M or 10 M). The solution was vortexed for 30 s with 250 µL of ethyl acetate and centrifuged at 20817 × *g* and 22 °C for 5 min. If whole-cell assays were extracted, the mixture was vortexed six times over 3 h. 200 µL of the organic phase was transferred into a new vial with 10 µL of HCl (2 M) and the mixture was vortexed for 20 s. Subsequently, the solvent was removed under reduced pressure for 20 min with a Concentrator 5301 (Eppendorf, Hamburg, Germany). The residue was dissolved in 100 µL methanol and undissolved solids were removed by membrane filtration with a PVDF filter (0.2 µm, ISERA GmbH, Düren, Germany) through centrifugation at 1228 × *g* for 2 min. The flowthrough was used for HPLC-MS/MS analysis.

Method B

For alkaloid extraction via method B, the pH of 200 µL of lysate was adjusted using 10 µL of HCl (2 M). The solution was vortexed for 30 s with 250 µL of ethyl acetate and centrifuged at 20817 × *g* and 22 °C for 5 min. The aqueous phase was transferred into a new vial and the pH was adjusted using 10 µL of NaOH (10 M). The mixture was vortexed for 30 s with 250 µL of ethyl acetate and centrifuged at 20817 × *g* and 22 °C for 5 min. 200 µL of the organic phase was transferred into a new vial and the second extraction step was repeated once. The organic phases were combined with 10 µL of HCl (2 M) and vortexed for 20 s. The solvent was removed under reduced pressure for 30 min with a Concentrator 5301 (Eppendorf, Hamburg, Germany). The residue was dissolved in 100 µL methanol and undissolved solids were removed by membrane filtration with a PVDF filter (0.2 µm, ISERA GmbH, Düren, Germany) through centrifugation at 1228 × *g* for 2 min. The flowthrough was used for HPLC-MS/MS analysis.

1.3 Reactions with Plant Lysate

AOS Activity Assays

AOS activity assays with plant lysate were performed in plant lysis buffer A (Table S6) in a 200 µL reaction at 37 °C and 300 rpm for 24 h (Thermomixer, Eppendorf, Hamburg, Germany). Substrates were added as solutions. Alanine derivatives were dissolved in the respective buffer just before activity assays. The concentration of substrate stock solutions and their pipetting scheme for the assay are shown in Table S7 and Table S8. Heat inactivation was performed by incubating the lysate samples at 95 °C and 300 rpm for 20 min. Negative control reactions were performed using heat-inactivated lysate, without the addition of lysate, and with missing substrates. After the assay, the reaction mixtures were extracted and analyzed via HPLC-MS/MS. The product formation was assessed using the retention time and the fragmentation pattern.

Table S7. Composition of stock solutions for AOS activity assays with plant assays.

Compound	Solvent	Concentration [mM]
L-Alanine	lysis buffer	250
[2,3,3,3-D_4]-L-Alanine	lysis buffer	250
^{15}N-L-Alanine	lysis buffer	250
Benzoyl-CoA	$_{dd}H_2O$	100

Table S8. Pipetting scheme for AOS activity assays with plant lysate.

Compound	Volume [µL]
Alanine derivative	40
Benzoyl-CoA	10
Crude lysate/<br>Lysate purified 1x via SEC/<br>Lysate purified 2x via SEC	150
Lysis buffer	to 200

2 Fragmentation Pattern of (*S*)-Cathinone

The characteristic fragmentation pattern and the *m*/*z* of the resulting ions of (*S*)-**1** in HPLC-MS/MS were identified using a standard reference. Corresponding structures were proposed based on literature.[12–14] For (*S*)-^{15}N-**1**, no standard reference was obtained and the detection method was manually adjusted with respect to the increased *m*/*z* of M+1 of the MRM transitions that are expected to contain the labeled nitrogen.

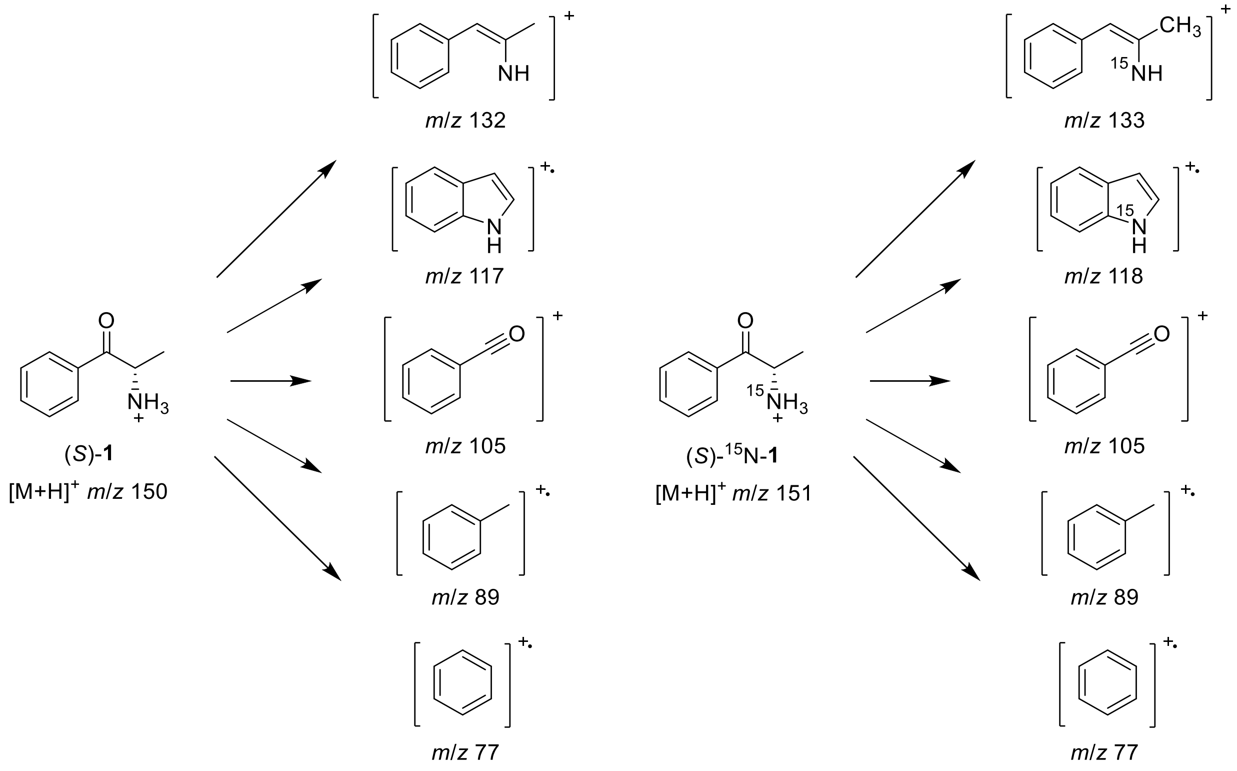

Scheme S1. The precursor ions (*S*)-cathinone [(*S*)-1] and (*S*)-^{15}N-cathinone [(*S*)-^{15}N-1], and the structures of their proposed fragment ions expected in mass spectrometry.

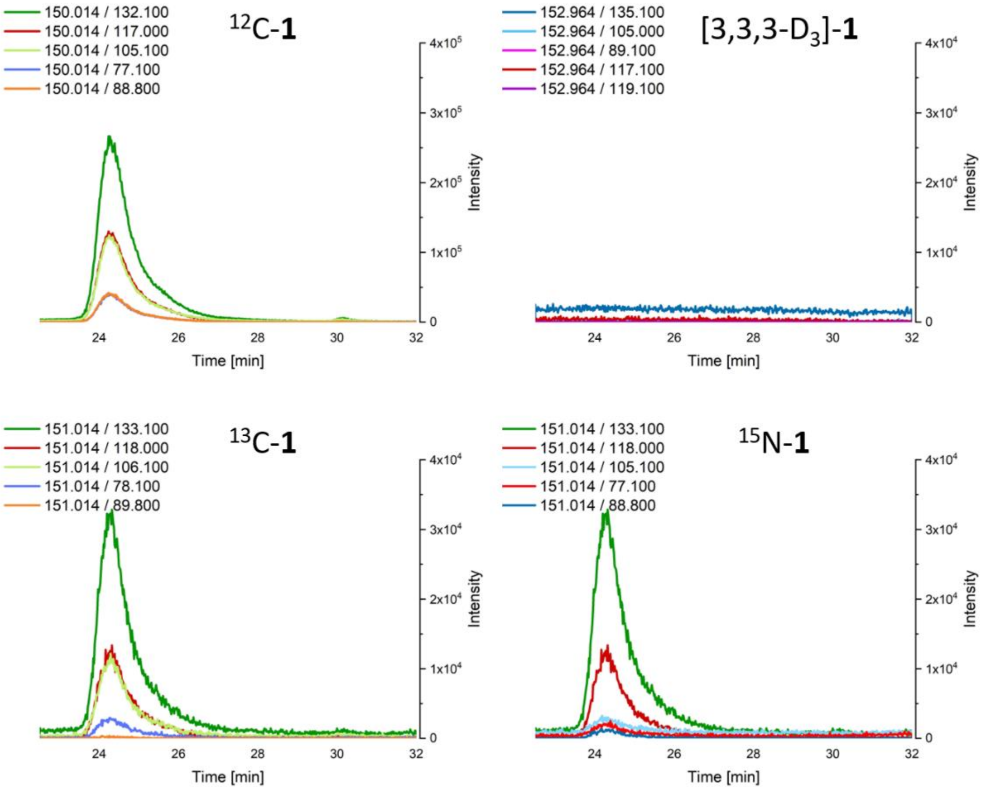

Figure S1. (*S*)-Cathinone [(*S*)-1] used as reference in HPLC-MS/MS. The chromatograms show the isolated ion transitions from the MRM scans expected for (*S*)-1 (top left), (*S*)-[3,3,3-D_3]-1 (top right), (*S*)-^{13}C-1 (bottom left), and (*S*)-^{15}N-1 (bottom right).

3 Possible Incorporation of Deuterium Label via Transamination

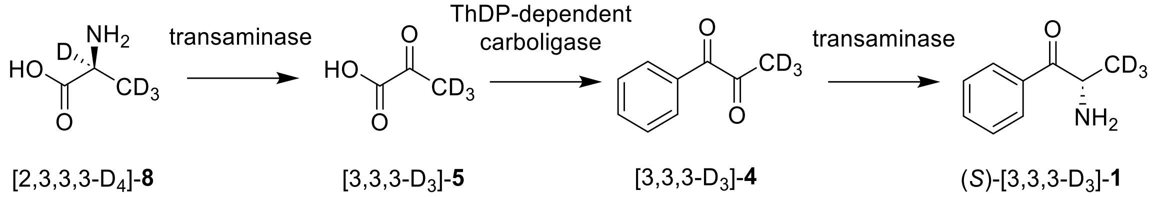

Scheme S2. Possible incorporation of label from [2,3,3,3-D4]-L-alanine [[2,3,3,3-D4]-8] into (*S*)-[3,3,3-D3]-cathinone [(*S*)-[3,3,3-D3]-1] via transamination to [3,3,3-D3]-pyruvate [[3,3,3-D3]-4].

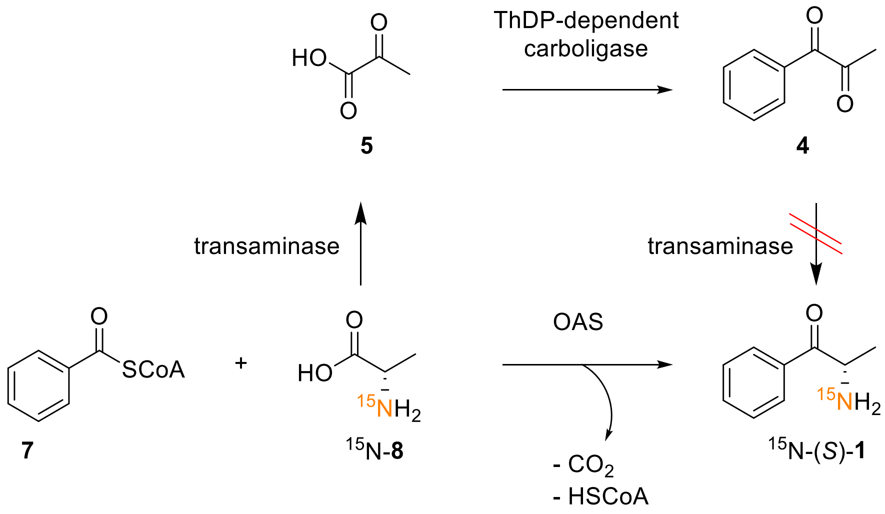

Scheme S3. The incorporation of the nitrogen label from ^{15}N-L-alanine [^{15}N-8] into (*S*)-^{15}N-cathinone [(*S*)-^{15}N-1] via an AOS bypassing the intermediates pyruvate (5) and 1-phenylpropane-1,2-dione (4).

4 Ephedra Alkaloid Content in tested Plants

Table S9. Ephedra alkaloids present in young stems of the six *Ephedra* species obtained from the Botanical Garden of Freiburg. The analyses were performed in April and May 2020 at the Institute of Pharmaceutical Sciences. The ratio of stereoisomers was calculated by comparing the AUC of the different peaks in 2-3 analyses and taking the average.

***Ephedra* Species**	**(1*S*,2*R*)-Norephedrine**	**(1*S*,2*S*)-Norpseudoephedrine**	**(1*R*,2*S*)-Norephedrine**	**(1*R*,2*S*)-Ephedrine**	**(1*S*,2*S*)-Pseudoephedrine**	**(1*R*,2*S*)-*N*-Methylephedrine**	**(1*S*,2*S*)-*N*-Methylpseudoephedrine**	**(*S*)-Cathinone**	**(*R*)-Cathinone**	**(*R/S*)-*N*-Methylcathinone**	**(*S/R*)-*N*-Methylcathinone**
E. americana var. andina	(+)	+	+	+	+	+	n.d.	+	+	n.d.	+
	1 : 28 : 72			71 : 29				86 : 14			
E. gerardiana var. gerardiana	(+)	+	+	+	+	+	+	+	+	+	+
	1 : 64 : 35			29 : 71		87 : 13		77 : 23		7 : 93	
E. intermedia	(+)	+	+	+	+	+	+	+	+	+	+
	<0.5 : 69 : 31			10 : 90		88 : 12		76 : 24		5 : 95	
E. fedtschenkoi	n.d.	n.d.	n.d.	n.d.	n.d.	n.d.	n.d.	n.d.	n.d.	n.d.	n.d.
E. major subsp. procera	n.d.	n.d.	n.d.	(+)	(+)	n.d.	n.d.	n.d.	n.d.	n.d.	n.d.
				67 : 33							

\+ Peaks are clearly detectable;
(+) Traces or peaks detected only in some analyses;
n.d. Peaks were not detected in any analysis.

Table S10. Ephedra alkaloids present in young tissue samples of *Ephedra* species obtained from different botanical gardens. The ratio of stereoisomers was calculated by comparing the AUC of the different peaks in a single analysis.

***Ephedra* Species**	**(1*S*,2*R*)-Norephedrine**	**(1*S*,2*S*)-Norpseudoephedrine**	**(1*R*,2*S*)-Norephedrine**	**(1*R*,2*S*)-Ephedrine**	**(1*S*,2*S*)-Pseudoephedrine**	**(1*R*,2*S*)-*N*-Methylephedrine**	**(1*S*,2*S*)-*N*-Methylpseudoephedrine**	**(*S*)-Cathinone**	**(*R*)-Cathinone**	**(*R/S*)-*N*-Methylcathinone**	**(*S/R*)-*N*-Methylcathinone**
E. chilensis (Botanical Garden of Bonn, 11.05.2022)	n.d.	n.d.	n.d.	n.d.	n.d.	n.d.	n.d.	n.d.	n.d.	n.d.	n.d.
E. distachya (female) (Botanical Garden of Frankfurt, 11.05.2022)	+	+	(+)	+	+	+	+	+	+	+	+
	57 : 39 : 4			1 : 99		1 : 99		95 : 5		31 : 69	
E. distachya (Botanical Garden of Frankfurt, 11.05.2022)	n.d.	+	+	+	+	+	+	+	+	+	+
	<0.5 : 51 : 49			1 : 99		2 : 98		96 : 4		2 : 98	
E. distachya ssp. helvetica (Botanical Garden of Frankfurt, 11.05.2022)	n.d.	n.d.	n.d.	+	+	n.d.	n.d.	(+)	n.d.	n.d.	n.d.
				59 : 41							
E. likiangensis (Botanical Garden of Frankfurt, 11.05.2022)	n.d.	+	+	+	+	+	+	+	+	+	+
	<0.5 : 4 : 96			98 : 2		98 : 2		86 : 14		6 : 94	
E. monosperma (Botanical Garden of Frankfurt, 11.05.2022)	n.d.	n.d.	n.d.	+	(+)	(+)	n.d.	+	n.d.	n.d.	n.d.
				91 : 9							
E. chilensis (Palm Gardens Frankfurt, 11.05.2022)	n.d.	+	+	+	+	+	+	+	+	n.d.	+
	<0.5 : 10 : 90			85 : 15		74 : 26		94 : 6			
E. minuta (Palm Gardens Frankfurt, 11.05.2022)	+	+	+	+	+	+	+	+	+	+	+
	7 : 24 : 69			48 : 52		51 : 49		94 : 6		11 : 89	
E. sinica (Botanical Garden Konstanz, 30.05.2022)	n.d.	n.d.	n.d.	n.d.	n.d.	n.d.	n.d.	n.d.	n.d.	n.d.	n.d.

\+ Peaks are clearly detectable;
(+) Traces or peaks detected only in some analyses;
n.d. Peaks were not detected in any analysis.

Table S11. Ephedra alkaloids present in young leave tissue of *Catha edulis*. The ratio of stereoisomers was calculated by comparing the AUC of the different peaks of an analysis.

Species	(1*S*,2*R*)-Norephedrine	(1*S*,2*S*)-Norpseudoephedrine	(1*R*,2*S*)-Norephedrine	(1*R*,2*S*)-Ephedrine	(1*S*,2*S*)-Pseudoephedrine	(1*R*,2*S*)-*N*-Methylephedrine	(1*S*,2*S*)-*N*-Methylpseudoephedrine	(*S*)-Cathinone	(*R*)-Cathinone	(*R*/*S*)-*N*-Methylcathinone	(*S*/*R*)-*N*-Methylcathinone
Catha edulis, young leaves (Bern)	n.d.	+	+	n.d.	n.d.	n.d.	n.d.	+	+	n.d.	n.d.
	0 : 75 : 25							97 : 3			

+ Peaks are clearly detectable;
(+) Traces;
n.d. Peaks were not detected in any analysis.

5 Formation of (*S*)-Cathinone [(*S*)-1] in Activity Assay

Table S12. AOS activity tested in *Ephedra* species after February 2022.

***Ephedra* Species**	**L-alanine + benzoyl-CoA**			**[2,3,3,3-D_4]-L-alanine + benzoyl-CoA**			**^{15}N-L-alanine + benzoyl-CoA**		
	(*S*/*R*)-Cathinone	(*S*/*R*)-[3,3,3-D_3]-Cathinone	(*S*/*R*)-^{15}N-Cathinone	(*S*/*R*)-Cathinone	(*S*/*R*)-[3,3,3-D_3]-Cathinone	(*S*/*R*)-^{15}N-Cathinone	(*S*/*R*)-Cathinone	(*S*/*R*)-[3,3,3-D_3]-Cathinone	(*S*/*R*)-^{15}N-Cathinone
E. chilensis (Botanical Garden of Bonn, 11.05.2022)	n.d.	n.d.	n.d.	n.d.	n.d.	n.d.	n.d.	n.d.	n.d.
E. distachya (female) (Botanical Garden of Frankfurt, 11.05.2022)	(+)	n.d.	n.d.	n.d.	(+)	n.d.	n.d.	n.d.	+
E. distachya (Botanical Garden of Frankfurt, 11.05.2022)	+	n.d.	n.d.	n.d.	+	n.d.	n.d.	n.d.	+
E. distachya ssp. helvetica (Botanical Garden of Frankfurt, 11.05.2022)	n.d.	n.d.	n.d.	n.d.	n.d.	n.d.	n.d.	n.d.	n.d.
E. likiangensis (Botanical Garden of Frankfurt, 11.05.2022)	+	n.d.	n.d.	n.d.	+	n.d.	n.d.	n.d.	+
E. monosperma (Botanical Garden of Frankfurt, 11.05.2022)	+	n.d.	n.d.	n.d.	(+)	n.d.	n.d.	n.d.	+
E. chilensis (Palm Gardens Frankfurt, 11.05.2022)	+	n.d.	n.d.	n.d.	+	n.d.	n.d.	n.d.	+
E. minuta (Palm Gardens Frankfurt, 11.05.2022)	+	n.d.	n.d.	n.d.	(+)	n.d.	n.d.	n.d.	+
E. americana var. Andina (Botanical Garden of Freiburg, April-June 2022)	+	n.d.	n.d.	+	+	n.d.	n.d.	n.d.	+
	n.d.	n.d.	n.d.	n.d.	n.d.	n.d.	n.d.	n.d.	n.d.

E. fedtschenkoi (Botanical Garden of Freiburg, April-June 2022)									
E. gerardiana var. Gerardiana (Botanical Garden of Freiburg, April-June 2022)	+	n.d.	n.d.	(+)	+	n.d.	n.d.	n.d.	+
E. intermedia (Botanical Garden of Freiburg, April-June 2022)	+	n.d.	n.d.	(+)	+	n.d.	n.d.	n.d.	+
E. major subsp. procera (Botanical Garden of Freiburg, April-June 2022)	n.d.	n.d.	n.d.	n.d.	n.d.	n.d.	n.d.	n.d.	n.d.
E. sinica (Botanical Garden Konstanz, 30.05.2022)	(+)	n.d.	n.d.	n.d.	n.d.	n.d.	n.d.	n.d.	(+)

\+ Peaks are clearly detectable;
(+) Traces or small peaks;
n.d. Peaks were not detected in the analysis.

6 PLP-dependence of (*S*)-Cathinone Formation in Plant Lysate

To test PLP dependence of (*S*)-cathinone [(*S*)-**1**] formation in plant lysate, activity assays were performed in lysis buffer A with and without PLP (27.7 mg/L). Plant samples were lysed and purified via SEC in parallel in buffer with and without PLP. Stock solutions were prepared in buffer with and without buffer, respectively. The test series and the presence of PLP in used buffer is shown in Table S13.

Table S13. Test series for the investigation on PLP dependence of (*S*)-cathinone formation in plant lysate. PLP was added to a concentration of 27.7 mg/L.

Test series	Lysis and purification	Substrate solutions and buffer
A	with PLP	with PLP
B	without PLP	without PLP
C	without PLP	with PLP

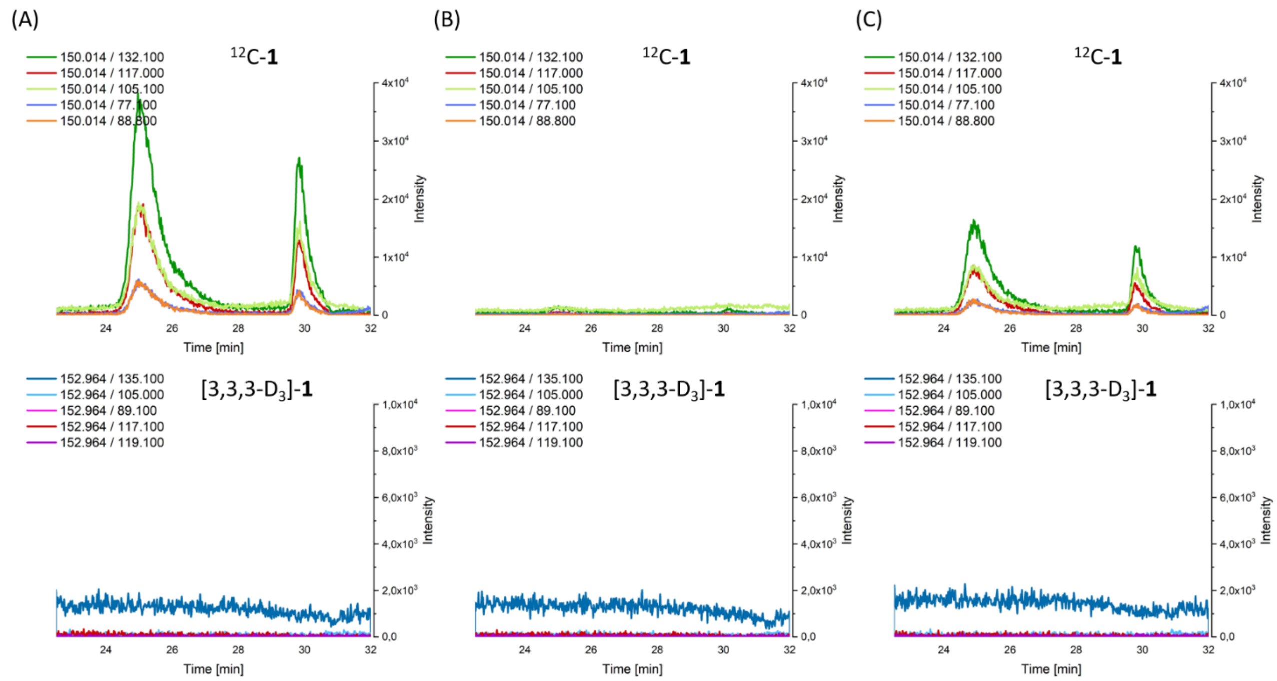

Figure S2. HPLC-MS/MS results of the three activity assays performed on lysate from *E. americana* collected in early June 2022 with benzoyl-CoA (7) and L-alanine (8). The lysis buffer used for size exclusion chromatography and the activity assays was (A) with PLP; (B) without PLP; (C) without PLP, but PLP was added just before the activity assay. The chromatograms show the isolated ion transitions from the MRM scans expected for (*S*)-1 (top) and (*S*)-[3,3,3-D$_3$]-1 (bottom).

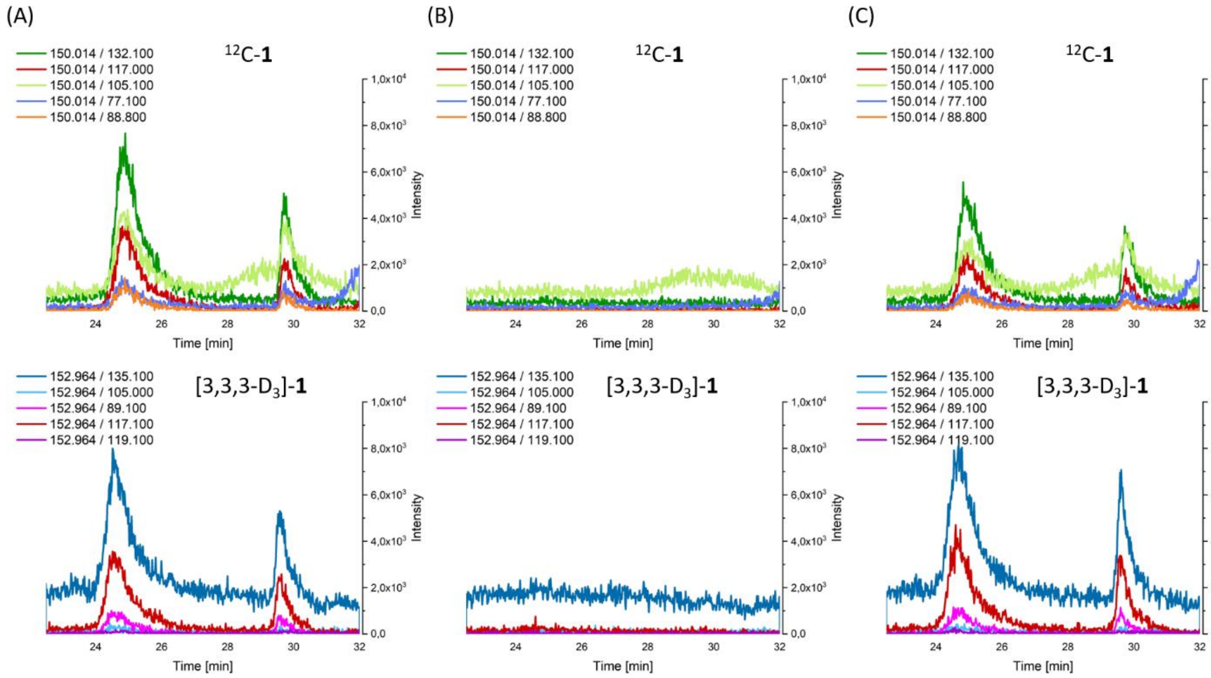

Figure S3. HPLC-MS/MS results of the three activity assays performed on lysate from *E. americana* collected in early June 2022 with 7 and [2,3,3,3-D_4]-8. The lysis buffer used for size exclusion chromatography and the activity assays was (A) with PLP; (B) without PLP; (C) without PLP, but PLP was added just before the activity assay. The chromatograms show the isolated ion transitions from the MRM scans expected for (*S*)-1 (top) and (*S*)-[3,3,3-D_3]-1 (bottom).
